# Preclinical efficacy and safety of drug-loaded porous silicon nanoparticles for cancer treatment: a systematic review of *in vitro* and *in vivo* studies

**DOI:** 10.3389/fphar.2026.1969890

**Published:** 2026-09-17

**Authors:** Cristian Sandoval-Vásquez, César Gatica, Sarahana Mendez, Valeria Oporto, Valentina Barra, Francisca Villagrán-Silva, Edgar Vásquez-Carrasco, Jordan Hernandez-Martinez, Laura Ochoa-Martinez, Schilin Wen, Paulina Sepúlveda-Figueroa

**Affiliations:** 1 Departamento de Medicina Interna, Facultad de Medicina, Universidad de La Frontera, Temuco, Chile; 2 Carrera de Tecnología Médica, Facultad de Medicina, Universidad de La Frontera, Temuco, Chile; 3 Magíster en Terapia Física con Mención, Facultad de Medicina, Universidad de La Frontera, Temuco, Chile; 4 Facultad de Ciencias de la Rehabilitación y Calidad de Vida, Universidad San Sebastián, Santiago, Chile; 5 School of Occupational Therapy, Faculty of Psychology, Universidad de Talca, Talca, Chile; 6 Centro de Investigación en Ciencias Cognitivas, Facultad de Psicología, Universidad de Talca, Talca, Chile; 7 VITALIS Longevity Center, Universidad de Talca, Talca, Chile; 8 Department of Physical Activity Sciences, Universidad de Los Lagos, Osorno, Chile; 9 Department of Education, Faculty of Humanities, Universidad de La Serena, La Serena, Chile; 10 Escuela de Tecnología Médica, Facultad de Salud, Universidad Santo Tomás, Osorno, Chile; 11 Grupo de Investigación de Pregrado en Odontología, Universidad Autónoma de Chile, Temuco, Chile; 12 Departamento de Ciencias Preclínicas, Facultad de Medicina, Universidad de La Frontera, Temuco, Chile

**Keywords:** biocompatibility, cancer therapy, drug delivery, functionalization, porous silicon nanoparticles

## Abstract

**Introduction:**

Porous silicon nanoparticles (pSiNPs) are promising carriers for anticancer therapy because of their high drug-loading capacity, modifiable surface properties, and potential for stimulus-responsive drug release. This systematic review assessed the efficacy and safety of drug-loaded pSiNPs in in vitro and *in vivo* cancer models.

**Methods:**

Searches were conducted from database inception to 30 July 2026. Eligible studies were controlled original investigations evaluating pSiNPs loaded with at least one anticancer drug and reporting quantitative antitumor outcomes. Study selection followed PRISMA 2020 guidelines, and the protocol was registered in PROSPERO (CRD420251076592). Given the substantial methodological heterogeneity, findings were synthesized narratively. *In vitro* study reliability was appraised using an explicitly adapted ToxRTool framework, whereas animal-study risk of bias was assessed with the SYRCLE tool.

**Results:**

The search strategy identified 974 records, of which 22 met the inclusion; eight included animal experiments. Targeting ligands, polymeric coatings, and pH-, redox-, magnetic-, or near-infrared-responsive systems generally improved cellular uptake, drug release, cytotoxicity, and antitumor activity relative to study-specific comparators. Most *in vitro* studies were classified as reliable with restrictions, while several animal-study domains had unclear risk of bias because key safeguards were insufficiently reported.

**Conclusion:**

Drug-loaded pSiNPs show promising preclinical anticancer activity, but the evidence is dominated by *in vitro* proof-of-concept studies. Animal evidence remains limited and does not establish long-term systemic safety or biocompatibility. Standardized nanoparticle characterization, rigorous animal designs, and comprehensive long-term safety and biodistribution studies are required before clinical translation.

**Systematic Review Registration:**

https://www.crd.york.ac.uk/PROSPERO/view/CRD420251076592.

## 1 Introduction

Cancer is a chronic non-communicable disease, and a large number of deaths worldwide are attributed to it (Parra-Soto et al., 2020). Cancer is one of the main causes of death worldwide, accounting for roughly 10 million deaths in 2020. About 20% of the population may be affected by cancer during their lifetime. An estimated 20 million new cases were reported worldwide in 2022 (Ferlay et al., 2021). There are more than 35 million new cases anticipated by 2050, a 77% rise from 2022 (World Health Organization, 2024). The disease originates with malignant alteration of normal cells and is a multi-step process that often includes progression from premalignant lesions to aggressive tumors. Cancer is the second leading cause of death worldwide, after cardiovascular disorders. It was estimated in 2020 that one in nine men and one in 12 women died of cancer-related causes (Ferlay et al., 2021; World Health Organization, 2025).

Some chronic infections are risk factors for cancer, a particular problem in low- and medium-income nations. In 2018, carcinogenic infections, including *Helicobacter pylori*, human papillomavirus (HPV), hepatitis B virus, hepatitis C virus and Epstein-Barr virus, contributed to around 13% of malignancies diagnosed worldwide (de Martel et al., 2020).

The increase of cancer incidence is closely associated with the increased chance of aberrant cellular proliferation with the aging of the population (Sandoval et al., 2018). Therefore, the development of new drug delivery techniques is important for enhancing treatment efficacy and reducing unwanted effects associated with conventional drugs, thereby improving patients' quality of life (Parra-Soto et al., 2020).

Emerging delivery platforms have received much attention by porous silicon nanoparticles (pSiNPs) and their structural variants due to their unique physicochemical features. These nanoparticles can be chemically functionalized to change optical, electrical and surface properties that make them useful in biosensing, targeted drug administration, tumor imaging, eye disease treatment and tissue engineering (Belogorlov et al., 2021). They provide a diverse platform for regulated drug release due to their biocompatibility, programmable degradation rates, large pore volume and tunable pore size (Qin et al., 2014). They are also stable, low-density, and biodegradable, and their size and shape can be controlled, making them suitable for individualized therapeutic designs. In addition, drug release can be controlled by environmental stimuli such as pH, temperature, light, magnetic fields, or electrical signals (Fang et al., 2023).

The therapeutic potential of pSiNP-based systems lies in their capacity to localize and control drug delivery, with the aim of improving anticancer efficacy while reducing off-target exposure (Deng et al., 2025). Further work is required to define their pharmacokinetics, biodistribution, degradation, cytotoxicity, and cellular uptake mechanisms. Accordingly, this review aimed to evaluate the preclinical efficacy and safety of drug-loaded pSiNPs in *in vitro* and *in vivo* cancer models.

## 2 Article types

### 2.1 Protocol and registration

This systematic review evaluated the preclinical efficacy of drug-loaded pSiNPs for cancer treatment using evidence from *in vitro* and *in vivo* experimental models. The review was conducted and reported in accordance with the Preferred Reporting Items for Systematic Reviews and Meta-Analyses (PRISMA 2020) statement (Page et al., 2021). Its protocol was prospectively registered in the International Prospective Register of Systematic Reviews (PROSPERO; CRD420251076592) (Vásquez and Sandoval, 2025).

### 2.2 Eligibility criteria

Eligibility criteria were established according to the PICOS framework (Population, Intervention, Comparator, Outcomes, and Study design), as detailed in Table 1.

**TABLE 1:** PICOS elegiblity criteria used in the systematic review.

Criteria	Inclusion criteria	Exclusion criteria
Population/model	Mammalian cancer cell lines, patient-derived cancer cells, three-dimensional tumor models, organoids, and mammalian animal models of cancer used to evaluate drug-loaded pSiNPs. Non-malignant mammalian cells were eligible when included as controls for assessing selectivity, cytocompatibility, or systemic toxicity	Studies exclusively involving non-cancerous cells or tissues without a cancer-related experimental model; non-mammalian models; studies without biological evaluation; and human clinical studies
Intervention	Porous elemental silicon (Si) nanoparticles loaded with at least one pharmacologically active anticancer drug. Studies using the terms porous, nanoporous, or mesoporous silicon were eligible when composition/characterization confirmed an elemental-Si porous framework. Co-delivery of an anticancer drug and a nucleic acid was eligible. Surface-functionalized, targeted, polymer-coated, stimulus-responsive, and hybrid formulations were eligible when porous elemental silicon remained the drug-carrying phase	Empty pSiNPs without a loaded anticancer drug; nonporous silicon particles; nucleic-acid therapy without an anticancer drug; mesoporous silica nanoparticles; non-silicon-based nanocarriers; silicon-containing composites in which porous elemental silicon was not the drug-carrying phase; microparticles; and macroscopic porous-silicon systems
Comparison	Untreated or vehicle-treated controls, free non-encapsulated therapeutic agents, empty pSiNPs, non-targeted nanoparticles, alternative nanocarriers, or baseline measurements	Studies without a free-drug, vehicle, empty-particle, untreated, or alternative-formulation comparator were excluded when therapeutic effectiveness attributable to the drug-loaded pSiNP formulation could not be distinguished
Outcomes	Primary outcomes included cancer cell viability or cytotoxicity, apoptosis or other forms of regulated cell death, tumor growth or tumor-volume inhibition, tumor burden, metastasis, and animal survival. Secondary outcomes included cellular uptake, intracellular drug delivery, biodistribution, pharmacokinetics, biocompatibility, systemic or organ toxicity, therapeutic selectivity, and drug-release behavior. Physicochemical characteristics, including particle size, morphology, surface charge, drug-loading capacity, encapsulation efficiency, and release profile, were extracted as formulation-related variables	Studies reporting only nanoparticle synthesis, physicochemical characterization, drug release, cellular uptake, biodistribution, imaging, photothermal effects without a loaded therapeutic agent, or biocompatibility without quantitative antitumor efficacy
Study design	Peer-reviewed original experimental studies employing controlled *in vitro*, or *in vivo* cancer models and reporting original quantitative data	Reviews, systematic reviews, meta-analyses, protocols, editorials, letters, conference abstracts, book chapters, case reports, *ex vivo* models, commentaries, and studies lacking original quantitative data

pSiNPs: porous silicon nanoparticles.

Particular attention was given to the classification of silicon-based nanomaterials because terminology in this field is not always used consistently across primary studies. Eligibility was therefore determined according to the chemical composition of the porous framework rather than solely on the basis of descriptors such as “porous,” “nanoporous,” or “mesoporous.” Studies evaluating nanoparticles composed of porous elemental silicon (Si) as the drug-carrying phase were eligible, irrespective of which of these terms was used by the original authors. Conversely, studies of mesoporous silica nanoparticles (SiO_2_), nonporous silicon particles, silicon-containing composites in which porous elemental silicon did not constitute the drug-carrying phase, microparticles, and macroscopic porous-silicon constructs were excluded. For reports in which the material terminology was unclear or potentially inconsistent, eligibility was determined by examining the reported composition together with the synthesis procedure and physicochemical characterization of the nanomaterial. This approach was used to distinguish porous elemental silicon from silica-based materials and to ensure consistent application of the eligibility criteria across studies.

### 2.3 Information sources and search strategy

A comprehensive literature search was conducted in MEDLINE, Embase, Scopus, Web of Science, International Pharmaceutical Abstracts, and Applied Science and Technology Source Ultimate from database inception to 30 July 2026. No language or publication-date restrictions were applied, and no study-design filter was used. Potentially eligible studies published in languages other than English were assessed using available translations. For reproducibility, Supplementary Table S1 reports, for each database, the platform/interface, date searched, exact line-by-line query as executed, fields searched, limits applied, and number of records retrieved before deduplication.

The search combined controlled vocabulary, where available, with free-text terms for three concepts: (1) porous elemental silicon nanoparticles, (2) drug loading/delivery, and (3) cancer. The core free-text logic was: (“porous silicon” OR “nanoporous silicon” OR “porous silicon nanoparticle*” OR “pSi nanoparticle*” OR pSiNP*) AND (“drug load*” OR “drug deliver*” OR nanocarrier* OR “controlled release” OR “targeted deliver*” OR “stimuli-responsive” OR “therapeutic agent*”) AND (cancer* OR neoplasm* OR tumor* OR tumour* OR carcinoma* OR malignant* OR oncology*). Database-specific controlled vocabulary, field tags, truncation symbols, and proximity operators were adapted to each interface and are reproduced verbatim in Supplementary Table S1. Terms for experimental models were used only when required by a database-specific supplementary search and were not used to exclude otherwise eligible records at screening.

The reference lists of included studies and relevant reviews were manually searched, and forward citation searching was conducted for all included studies. All supplementary identification routes were documented separately from the database search results. Duplicate records were removed before screening.

### 2.4 Study selection

All records retrieved from the databases were imported into Rayyan (Ouzzani et al., 2016), a web-based platform for managing systematic reviews. Duplicate records were identified and removed before screening. Two reviewers independently screened the titles and abstracts against the predefined eligibility criteria. During this stage, each reviewer was blinded to the other’s decisions to minimize selection bias.

Full-text reports were obtained for all records considered potentially eligible by either reviewer. The same reviewers independently assessed these reports and documented the reasons for exclusion. Disagreements at either stage were resolved through discussion and consensus. When consensus could not be reached, a third reviewer made the final decision.

The selection process was documented using a PRISMA 2020 flow diagram, which reports the numbers of records identified, duplicates removed, records screened, full-text reports assessed, and studies included in the review.

### 2.5 Supplementary study identification and research integrity checks

An independent expert was consulted to identify potentially relevant studies that might not have been retrieved through the electronic searches. The expert was required to (i) hold a doctoral degree in a health or biomedical science discipline and (ii) have peer-reviewed publications on drug-delivery systems, nanoparticles, or experimental cancer treatments. To minimize confirmation bias, the full electronic search strategy was not disclosed until the expert had completed the independent identification process.

Before the final synthesis, all included articles were checked for published corrections, expressions of concern, and retractions. This verification was completed on 30 July 2026, by reviewing the relevant bibliographic records, journal websites, and retraction databases. Retracted articles were excluded, while published corrections were considered during data extraction and interpretation.

### 2.6 Experimental models

Eligible *in vitro* models comprised established or patient-derived mammalian cancer cells, including drug-sensitive and drug-resistant variants, as well as three-dimensional tumor spheroids, organoids, blood–brain barrier models, and other biologically relevant multicellular cancer systems. Studies were eligible when these models were used to quantitatively evaluate the antitumor activity, cellular uptake, intracellular delivery, therapeutic selectivity, or mechanism of action of drug-loaded pSiNP formulations.

Non-malignant mammalian cells were eligible only when included as control models to assess cytocompatibility, treatment selectivity, inflammatory or immune responses, or off-target toxicity. Data obtained from these cells were considered safety or selectivity outcomes and were not treated as evidence of anticancer efficacy.

Eligible *in vivo* models included mammalian animals bearing experimentally induced, xenograft, orthotopic, syngeneic, metastatic, or genetically engineered tumors. No restrictions were imposed on animal species, strain, sex, or reproductive status, provided that the model was relevant to the cancer-treatment experiment. Healthy animals were eligible only when used as concurrent controls or for evaluating pharmacokinetics, biodistribution, biodegradation, biocompatibility, or systemic and organ-specific toxicity directly associated with the administration of a drug-loaded pSiNP formulation.

### 2.7 Data extraction

Data were extracted independently by two reviewers using a standard form, piloted *a priori*. The following data was collected: 1. Publication characteristics: authors, year of publication, country and funding sources; 2. Experimental model: kind of cancer; cell line or animal species and strain; sex, age, and body weight; method of tumor induction; and sample size; 3. Nanoparticles characteristics: pSiNP synthesis process, particle size, shape, porosity, surface charge, surface functionalization, targeting ligand and degrading properties; 4. Therapeutic formulation: drug or therapeutic agent, technique of loading, loading capacity, encapsulation efficiency, concentration or dose administered, route of administration, frequency of treatment and length of therapy; 5. Characteristics of comparator: untreated, vehicle-treated, free-drug, empty-particle, non-targeted-particle, or alternative-carrier controls; 6. Therapeutic outcomes: cell viability, half-maximal inhibitory concentration (IC_50_), apoptosis, cellular uptake, tumor volume, tumor-growth inhibition, metastatic burden, and survival; 7. Safety and distribution outcomes: cytocompatibility, biodistribution, pharmacokinetics, changes in body weight, hematological and biochemical markers, organ toxicity, and histopathological findings; and 8. Quantitative outcomes: means, measures of dispersion, effect estimates, confidence intervals, exact p-values, and sample sizes.

Any disagreements between the reviewers were resolved by consensus or, when necessary, consultation with a third reviewer.

### 2.8 Methodological quality and risk-of-bias assessment

Two conceptually distinct appraisal approaches were used. Methodological quality of *in vitro* studies was evaluated separately from risk of bias in animal studies. Reliability refers here to the completeness and adequacy of methodological reporting and the extent to which an *in vitro* result can be considered interpretable for the stated experimental conditions; it is not synonymous with risk of bias. Risk of bias refers to systematic deviations from the true intervention effect arising from study design, conduct, analysis, or reporting. All appraisals were performed independently by two reviewers, with disagreements resolved by consensus or, when necessary, by a third reviewer.

The SYRCLE risk of bias tool was used to assess the risk of bias for *in vivo* animal experiments. This tool addresses 10 domains of selection, performance, detection, attrition, reporting and other potential sources of bias. For each domain a rating of low, high or uncertain risk of bias was attributed and an aggregate number score was not calculated. Each element is rated as “yes”, “no” or “unclear” corresponding to low risk of bias, high risk of bias and insufficient detail to estimate the risk of bias, respectively (Hooijmans et al., 2014).

The reliability of the *in vitro* investigations was appraised using the *in vitro* module of the Toxicological Data Reliability Assessment Tool (ToxRTool) (Schneider et al., 2009). ToxRTool was selected because no single validated risk-of-bias instrument is established for the heterogeneous nanoparticle–cancer cell experiments included in this review, and because its *in vitro* module provides structured criteria for test-system identification, intervention characterization, experimental design, controls, exposure conditions, outcome assessment, data reporting, and biological plausibility. The tool was adapted only for applicability to drug-loaded pSiNP efficacy experiments: toxicology-specific wording was mapped to the nanoparticle intervention and cancer-cell test system, while the original 18-point structure and reliability thresholds were retained. No ToxRTool score was interpreted as a risk-of-bias judgment. Because this adaptation has not itself been independently validated for nanomedicine efficacy studies, the resulting categories were treated as a descriptive reliability appraisal and used to contextualize, rather than determine, the conclusions.

Studies were not rejected purely on the basis of methodological quality or risk-of-bias classification. Instead, these assessments served to inform the interpretation of the consistency, robustness and certainty of the available data.

### 2.9 Data synthesis

Characteristics and findings of the included studies were presented in structured tables and synthesized narratively. Data from *in vitro* and *in vivo* investigations were analyzed separately. Studies were categorized according to cancer type, therapeutic drug, pSiNP functionalization or targeting approach, experimental model, comparator and therapeutic effect.

The *in vitro* synthesis focused on cancer cell viability, cytotoxicity, IC_50_, apoptosis, cellular uptake, and selectivity relative to non-malignant cells. The *in vivo* synthesis focuses on tumor-growth inhibition, tumor burden, survival, biodistribution, systemic toxicity and histopathological results.

Substantial methodological heterogeneity precluded meta-analysis. Major sources of heterogeneity included differences in experimental models, nanoparticle formulations, payloads, comparators, doses, exposure periods, and outcome definitions. Accordingly, no pooled effect estimate was calculated, and a structured narrative synthesis was undertaken. Interpretation considered the magnitude and direction of reported findings where numerical data were available and was not based solely on statistical significance. The research selection procedure is represented in the PRISMA 2020 flow diagram (Figure 1).

**FIGURE 1:**
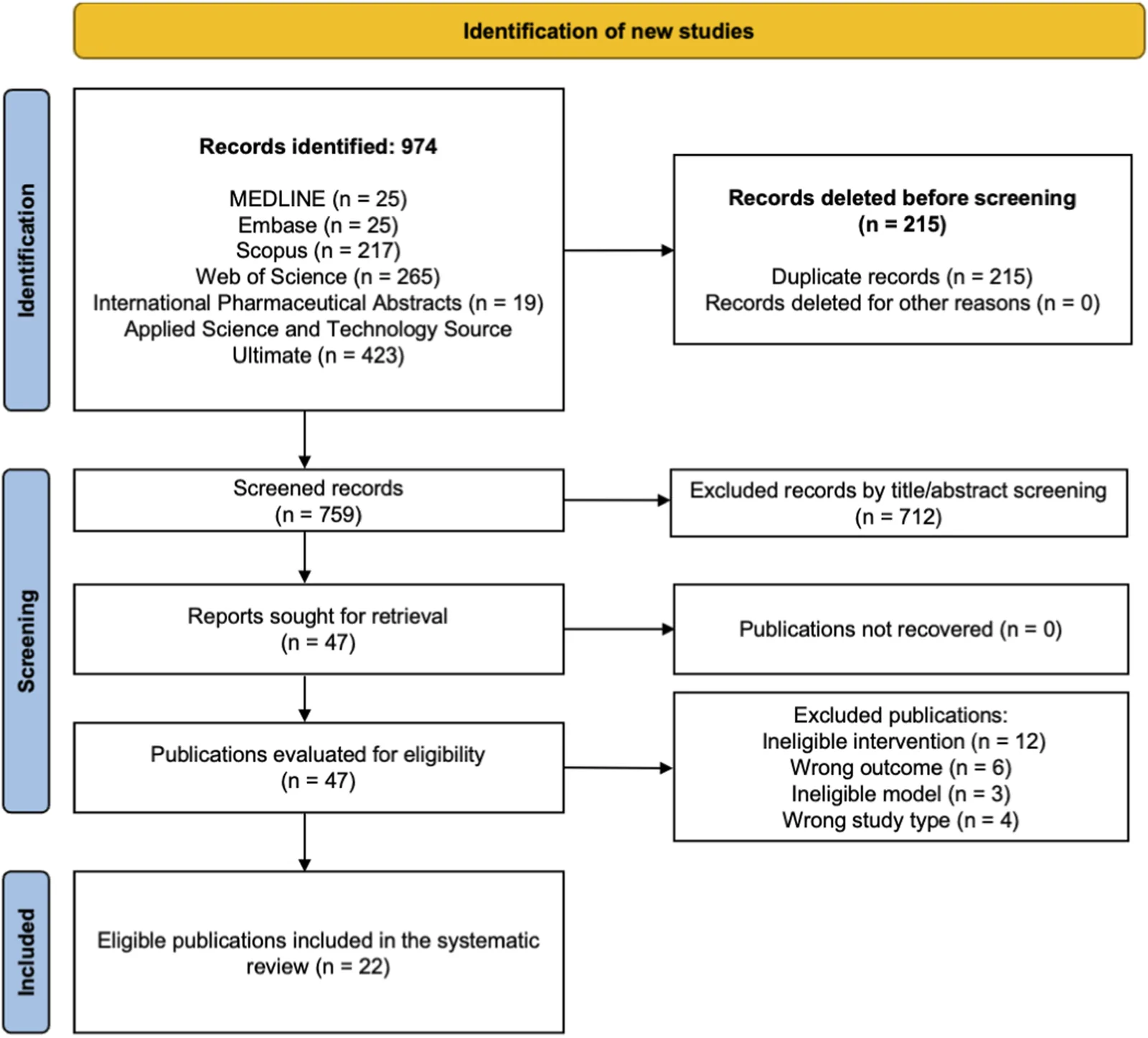
Flowchart of the systematic review.

To strengthen the quantitative synthesis, study-level effect magnitudes were extracted whenever they were explicitly reported or could be derived without assumptions, including percentage changes in cell viability, IC50 contrasts, cellular-uptake enhancement, tumor-volume or tumor-growth inhibition, metastatic burden, and survival. Values that were not reported or were not directly comparable because of different doses, exposure periods, assay definitions, or normalization procedures were classified as not reported/not extractable rather than imputed. Cross-study medians or pooled ranges were not calculated when the underlying measurements were not sufficiently commensurable.

## 3 Results

### 3.1 Study selection

The database searches yielded 974 records. After removal of 215 duplicates, 759 titles and abstracts were examined. Forty-seven publications in full text were assessed for eligibility, and 25 research were removed for the reasons shown in Figure 1. The most common reasons were use of nonporous silicon or mesoporous silica platforms, absence of a loaded anticancer drug, reporting of imaging or drug-release outcomes without eligible antitumor outcomes, use of microparticles or macroscopic porous-silicon systems, or absence of an eligible cancer model or comparator. Twenty-two original experimental investigations fulfilling all qualifying criteria were included in the qualitative synthesis. A meta-analysis was not conducted due to the significant heterogeneity of cancer models, pSiNP formulations, therapeutic payloads, comparators, treatment regimens and outcome measures.

### 3.2 Characteristics of the selected studies

The papers considered in this review were published from 2014 to 2026 and examined drug-loaded pSiNP platforms in a wide variety of experimental cancer types (Zhang et al., 2022; Correia et al., 2015; Abdelmoneem et al., 2025; Shahbazi et al., 2015; Konoplyannikov et al., 2021; Shahbazi et al., 2014; Shin et al., 2024; Wang et al., 2015; Luo et al., 2023; Landgraf et al., 2020; Tieu et al., 2021; Xia et al., 2018; Oaxaca Camacho et al., 2025; Zhang et al., 2017; Liu et al., 2017; Xue et al., 2022; Malhotra et al., 2025; Li et al., 2020; Tran et al., 2022; Luo et al., 2019; Zeng et al., 2019; Xie et al., 2018). Most of the investigations employed *in vitro* cancer cell lines, but some studies also used three-dimensional spheroids, blood-brain barrier models, xenografts, orthotopic tumors or syngeneic murine tumor models (Table 2) (Zhang et al., 2022; Konoplyannikov et al., 2021; Shin et al., 2024; Luo et al., 2023; Liu et al., 2017; Xue et al., 2022; Li et al., 2020; Tran et al., 2022; Zeng et al., 2019; Xie et al., 2018).

**TABLE 2:** Main characteristics and therapeutic outcomes of included studies.

Ref.	Cancer model	Cell line or animal species/strain	Formulation and payload	Model type	Reported sample size/replicates	Comparator(s)	Dose/concentration	Exposure or follow-up
(Zhang et al., 2022)	Prostate cancer	C4-2B cells; 2D cultures and 3D spheroids	CPT-loaded core–shell pSiNPs functionalized with polyHPAm and cRGDfK	*In vitro*	NR	Untreated cells; free CPT; empty and non-targeted pSiNPs	Multiple concentrations; exact range NR	NR
(Correia et al., 2015)	Breast cancer	Breast cancer cells; healthy rat kidney and liver cells as controls	Sorafenib-loaded cyclodextrin-modified pSiNPs	*In vitro*	Three replicates	Untreated cells; free sorafenib; empty pSiNPs	Multiple concentrations	8 and 24 h for selected assays
(Abdelmoneem et al., 2025)	Osteosarcoma	MG-63 and Saos-2 cells	DOX-loaded, alendronate-targeted and redox-responsive pSiNPs	*In vitro*	NR	Free DOX; empty pSiNPs; non-targeted DOX-pSiNPs	Multiple concentrations	NR
(Shahbazi et al., 2015)	Breast cancer	CD326-expressing breast cancer cells	Sorafenib-loaded, CD326 antibody-targeted pSiNPs	*In vitro*	NR	Untreated cells; free sorafenib; untargeted and empty pSiNPs	Multiple concentrations	8 and 24 h
(Konoplyannikov et al., 2021)	Breast and lung cancer	MCF-7 and MCF-7/MDR1 cells; Lewis lung carcinoma-bearing mice	Salinomycin-loaded mesoporous silicon nanoparticles	*In vitro* and *in vivo*	Five mice per group for lung histology; total group size NR	Untreated control; free salinomycin; empty nanoparticles	*In vivo*: 0.2 mL at 0.5 mg/mL, intraperitoneally	Every second day for 10 days; five administrations
(Shahbazi et al., 2014)	Breast cancer	MDA-MB-231 and MCF-7 cells	MTX-loaded pSiNPs with a zwitterionic bilayer polymer coating	*In vitro*	NR	Untreated cells; free MTX; empty and uncoated pSiNPs	Nanoparticles up to 100 μg/mL; exact MTX-equivalent doses NR	6 and 24 h
(Shin et al., 2024)	Glioblastoma	Glioblastoma cells; immunodeficient BALB/c nude mice bearing intracranial tumors	TMZ-loaded octadecyl-modified pSiNPs	*In vitro* and *in vivo*	NR	Untreated control; free TMZ; empty pSiNPs	Equivalent free-TMZ and nanoparticle doses; exact dose NR	NR
(Wang et al., 2015)	Breast cancer and glioblastoma	Breast cancer cells and U87-MG glioblastoma cells	Dual-drug-loaded pSiNPs containing sorafenib and MTX	*In vitro*	NR	Free drugs individually and combined; empty particles; single-drug formulations	Multiple concentrations	NR
(Luo et al., 2023)	Glioblastoma	U87-mCherry cells; hCMEC/D3 BBB model; orthotopic glioma-bearing mice	AON- and TMZ-loaded multifunctional pSiNPs	*In vitro*, BBB and *in vivo*	NR	Untreated cells; free AON/TMZ; empty pSiNPs; control oligonucleotide	TMZ loading approximately 1.3 mg per formulation batch; biological dose NR	NR
(Landgraf et al., 2020)	Breast cancer bone metastasis	MDA-MB-231BO cells; female NOD-scid IL2Rγ-null mice with humanized bone constructs	CPT-loaded, EGFR-targeted silicon nanoparticles	*In vitro* and *in vivo*	NR	Free CPT; non-targeted CPT-pSiNPs; empty particles; vehicle	*In vitro*: free CPT equivalent of 6.6 μg/mL in selected assays	60 h *in vitro*; *in vivo* follow-up NR
(Tieu et al., 2021)	Multidrug-resistant cancer	C4-2B, C32, MDA-MB-231BO and AT3 cancer cells; HEK293-WT controls	Nanobody-functionalized pSiNPs co-loaded with DOX and siRNA against MRP1	*In vitro*, including 3D spheroids	Four replicates for selected uptake assays	Untreated cells; PEG-pSiNPs; single-cargo and non-targeted formulations	Multiple concentrations	NR
(Xia et al., 2018)	Multidrug-resistant breast cancer	MCF-7 and MCF-7/ADR cells	pSiNPs co-loaded with a chemotherapeutic drug and photothermal agent	*In vitro*	NR	Untreated cells; free drug; drug-only and photothermal-only formulations	Multiple concentrations; NIR irradiation applied	NR
(Oaxaca Camacho et al., 2025)	HER2-positive breast cancer	HER2-positive SKBR3 cells and appropriate low-HER2 controls	HER2 antibody-functionalized, DOX-loaded nanoporous silicon particles	*In vitro*	NR	Free DOX; empty particles; non-functionalized DOX-pSiNPs	Multiple concentrations	NR
(Zhang et al., 2017)	Breast cancer	Breast cancer cells with receptor-expression controls	MTX-loaded pSiNPs with receptor-mediated surface-charge inversion	*In vitro*	NR	Free MTX; empty pSiNPs; non-responsive formulation	Multiple concentrations	6 h for selected uptake/viability experiments
(Liu et al., 2017)	Doxorubicin-resistant breast cancer	MCF-7 and DOX-resistant MCF-7 cells	Quercetin-modified, DOX-loaded pSiNPs	*In vitro*	NR	Untreated cells; free DOX; empty and unmodified DOX-pSiNPs	Multiple concentrations	NR
(Xue et al., 2022)	Cervical cancer	HeLa cells; female BALB/c nude mice bearing HeLa xenografts	DOX-loaded pSiNPs with stimulus-cleavable linkers	*In vitro* and *in vivo*	NR	PBS; free DOX; empty particles; non-activated and activated formulations	*In vitro*: up to 100 μg/mL particles; *in vivo*: 1 mg/kg DOX equivalent	48 h *in vitro*; *in vivo* schedule reported graphically
(Malhotra et al., 2025)	Triple-negative breast cancer	MCF10Ca1h cells; MCF10A controls; female BALB/c nude mice	Aptamer-targeted, DOX-loaded pSiNPs	*In vitro* and *in vivo*	Triplicate wells for selected assays; animal number NR	Untreated cells; free DOX; empty pSiNPs; antibody-targeted and non-targeted formulations	Particles: 50 μg/mL in selected assays	2–48 h *in vitro*; *in vivo* follow-up NR
(Li et al., 2020)	Multidrug-resistant breast cancer	MCF-7 and MDR/MCF-7 cells; 3D multicellular spheroids	DOX-loaded pSiNPs incorporating Fe_3_O_4_/Au components	*In vitro*	At least five replicates for viability assays	Free DOX; untreated cells; particles without NIR or magnetic activation	NIR: 1.6 W/cm^2^ for 10 min; DOX-equivalent concentrations varied	Additional 24 h after irradiation
(Tran et al., 2022)	Cervical cancer and neuroblastoma	HeLa and SH-SY5Y cells; HEK293T non-malignant controls	Cisplatin-loaded, graphene oxide-wrapped mesoporous silicon nanoparticles with mitochondrial targeting	*In vitro*	NR	Untreated cells; free cisplatin; empty particles; non-targeted formulation	Particles up to 100 μg/mL	24 h
(Luo et al., 2019)	Glioblastoma	U87-mCherry cells; hCMEC/D3 BBB model; HaCaT controls	Transferrin-functionalized, DOX-loaded pSiNPs	*In vitro* and BBB model	NR	Free DOX; empty pSiNPs; non-targeted DOX-pSiNPs	Particles: 100 μg/mL in selected uptake assays; DOX loading solution 200 μg/mL	3 h for selected uptake assays; other durations NR
(Zeng et al., 2019)	Glioma	NCH-421K and C6 cells; male BALB/c nude mice bearing subcutaneous C6 tumors	TMZ-loaded pSiNPs combined with mild photothermal therapy and HBO	*In vitro* and *in vivo*	Six treatment groups; animals per group NR	Control; empty PSi; free TMZ; PTT; TMZ/PSi; TMZ/PSi + PTT + HBO	*In vitro*: TMZ 50–1600 μM; *in vivo*: TMZ 5 mg/kg	24–72 h *in vitro*; treatment on days 1, 4, 7 and 10
(Xie et al., 2018)	Glioma	C6 cells; male BALB/c nude mice aged 6–8 weeks with C6 tumors	TMZ-loaded pSiNPs combined with HBO	*In vitro* and *in vivo*	Six treatment groups; animals per group NR	Model control; empty PSi; HBO; free TMZ; TMZ/PSi; TMZ/PSi + HBO	*In vitro*: TMZ 50–1600 μM; 400 µM for selected uptake assays	48 h under hypoxia for selected assays; animals followed to day 22

AON: antisense oligonucleotide; BBB: blood–brain barrier; CPT: camptothecin; DOX: doxorubicin; EGFR: epidermal growth factor receptor; HBO: hyperbaric oxygen; HER2: human epidermal growth factor receptor 2; MDR: multidrug resistance; MRP1: multidrug resistance-associated protein 1; MTX: methotrexate; NIR: near-infrared; NR: indicates that the exact value was not reported in an extractable numerical form in the primary article; it does not indicate that an available numerical value was omitted during review extraction; PEG: polyethylene glycol; polyHPAm: poly(N-(2-hydroxypropyl)methacrylamide); PSi: porous silicon; pSiNPs: porous silicon nanoparticles; PTT: photothermal therapy; siRNA: small interfering RNA; TMZ: temozolomide; TNBC: triple-negative breast cancer.

Breast cancer and glioblastoma were the most frequently investigated malignancies. Breast cancer models included MCF-7, MCF-7/ADR or other multidrug-resistant variants, MDA-MB-231, HER2-positive SKBR3 cells, and triple-negative breast cancer cells (Correia et al., 2015; Shahbazi et al., 2015; Shahbazi et al., 2014; Tieu et al., 2021; Xia et al., 2018; Oaxaca Camacho et al., 2025; Zhang et al., 2017; Liu et al., 2017; Xue et al., 2022; Malhotra et al., 2025; Li et al., 2020). Glioblastoma studies used U87-derived models, C6 cells, NCH-421K glioma stem-like cells, and intracranial or subcutaneous tumor-bearing mice (Shin et al., 2024; Luo et al., 2023; Luo et al., 2019; Zeng et al., 2019; Xie et al., 2018). Other models included prostate carcinoma, osteosarcoma, and Lewis lung carcinoma (Zhang et al., 2022; Abdelmoneem et al., 2025; Konoplyannikov et al., 2021).

The therapeutic payloads were diverse. Doxorubicin was the most commonly evaluated agent and was incorporated into pSiNPs alone or combined with siRNA, photothermal agents, targeting ligands, or stimuli-responsive coatings (Abdelmoneem et al., 2025; Tieu et al., 2021; Xia et al., 2018; Oaxaca Camacho et al., 2025; Liu et al., 2017; Xue et al., 2022; Malhotra et al., 2025; Li et al., 2020; Luo et al., 2019). Other small-molecule drugs included camptothecin (Zhang et al., 2022; Landgraf et al., 2020), sorafenib (Correia et al., 2015; Shahbazi et al., 2015; Wang et al., 2015), temozolomide (Shin et al., 2024; Luo et al., 2023; Zeng et al., 2019; Xie et al., 2018), methotrexate (Shahbazi et al., 2014; Wang et al., 2015; Zhang et al., 2017), salinomycin (Konoplyannikov et al., 2021), and cisplatin (Tran et al., 2022). A quercetin-modified doxorubicin formulation was also evaluated in drug-resistant breast cancer cells (Liu et al., 2017). Near-infrared-responsive and photothermal components were incorporated to enable controlled chemo-photothermal treatment (Xia et al., 2018; Xue et al., 2022; Li et al., 2020; Tran et al., 2022; Zeng et al., 2019).

Most formulations used surface engineering to improve tumor selectivity, circulation, intracellular delivery, or stimulus-responsive release. These strategies included antibodies against CD326 or HER2 (Shahbazi et al., 2015; Oaxaca Camacho et al., 2025), transferrin (Luo et al., 2019), nanobodies (Tieu et al., 2021), tumor-targeting aptamers (Malhotra et al., 2025), cRGDfK peptides (Zhang et al., 2022), alendronate (Abdelmoneem et al., 2025), quercetin derivatives (Liu et al., 2017), polyethylene glycol (Tieu et al., 2021), zwitterionic polymer layers (Shahbazi et al., 2014), and graphene oxide or iron oxide/gold-based hybrid components (Li et al., 2020; Tran et al., 2022). Across studies, pSiNPs generally acted as the primary porous cargo reservoir, while surface modifications were intended to enhance targeting, stability, endosomal escape, or controlled release (Table 3) (Zhang et al., 2022; Correia et al., 2015; Abdelmoneem et al., 2025; Shahbazi et al., 2015; Konoplyannikov et al., 2021; Shahbazi et al., 2014; Shin et al., 2024; Wang et al., 2015; Luo et al., 2023; Landgraf et al., 2020; Tieu et al., 2021; Xia et al., 2018; Oaxaca Camacho et al., 2025; Zhang et al., 2017; Liu et al., 2017; Xue et al., 2022; Malhotra et al., 2025; Li et al., 2020; Tran et al., 2022; Luo et al., 2019; Zeng et al., 2019; Xie et al., 2018).

**TABLE 3:** Main therapeutic outcomes and quantitative or comparative findings.

Ref.	Primary outcome(s)	Main quantitative or comparative finding
(Zhang et al., 2022)	Cellular association, CPT release and cell viability	Targeted CPT-pSiNPs produced greater cellular association and dose-dependent cytotoxicity than non-targeted particles in 2D cultures and 3D spheroids; exact numerical effect estimate NR
(Correia et al., 2015)	Drug release, viability and proliferation	Cyclodextrin modification provided sustained sorafenib release and greater inhibition of cancer-cell proliferation than empty particles; exact numerical effect estimate NR
(Abdelmoneem et al., 2025)	Cellular uptake and viability	The targeted redox-responsive formulation reduced MG-63 viability to approximately 30%, compared with approximately 50% viability for redox-responsive delivery without equivalent targeting
(Shahbazi et al., 2015)	Targeted association and antiproliferative activity	CD326 functionalization increased cancer-cell association and antiproliferative activity relative to untargeted formulations; exact numerical effect estimate NR
(Konoplyannikov et al., 2021)	Cell viability, tumor growth and survival	Salinomycin-loaded particles suppressed Lewis lung carcinoma growth more effectively than free salinomycin or empty particles; exact tumor-growth difference NR
(Shahbazi et al., 2014)	Cellular uptake, endosomal escape and viability	The polymer-coated formulation increased intracellular trafficking and endosomal escape relative to uncoated particles; exact numerical therapeutic effect NR
(Shin et al., 2024)	TMZ stability, brain accumulation, tumor burden and survival	TMZ-pSiNPs decreased intracranial tumor burden and prolonged survival compared with equivalent free-TMZ dosing; exact numerical survival estimates NR
(Wang et al., 2015)	Cellular uptake, sustained release and combination activity	Dual-drug delivery produced stronger antiproliferative activity than single-drug formulations; exact numerical effect estimate NR
(Luo et al., 2023)	BBB penetration, migration and antitumor activity	The multifunctional particles crossed the BBB, reduced migration and improved antitumor activity relative to control formulations; exact numerical therapeutic effect NR
(Landgraf et al., 2020)	Cell viability, primary tumor growth and metastatic burden	Targeted CPT-pSiNPs reduced primary tumor growth and breast cancer bone-metastatic involvement relative to free CPT and non-targeted particles; exact numerical effect estimates NR
(Tieu et al., 2021)	Cellular association, MRP1 downregulation and viability	PEG-pSiNPs associated with fewer than 8% of evaluated cells, whereas targeted particles reached approximately 84% cellular association in selected receptor-positive cells; co-delivery enhanced cytotoxicity
(Xia et al., 2018)	Viability and chemo-photothermal activity	Combined drug and photothermal delivery reduced viability of resistant breast cancer cells more strongly than chemotherapy or photothermal treatment alone; exact numerical effect estimate NR
(Oaxaca Camacho et al., 2025)	HER2-specific uptake and viability	Antibody-functionalized particles increased selective uptake and DOX cytotoxicity in HER2-positive cells relative to non-targeted particles; exact numerical effect estimate NR
(Zhang et al., 2017)	Cancer-cell recognition, uptake and viability	Receptor-mediated charge inversion increased cellular association and therapeutic activity compared with the non-responsive formulation; exact numerical effect estimates NR
(Liu et al., 2017)	Intracellular DOX accumulation and resistant-cell viability	Quercetin modification increased DOX accumulation and inhibition of DOX-resistant breast cancer cells relative to free DOX and unmodified particles; exact numerical effect estimate NR
(Xue et al., 2022)	Stimulus-responsive release, cell viability and tumor growth	Drug-release performance: approximately 82% at pH 5.2 versus 2.2% at pH 7.4 after 48 h. Therapeutic effect: more than 70% cell death at 100 μg/mL following activation
(Malhotra et al., 2025)	Targeted uptake, viability and tumor growth	Aptamer targeting increased delivery to TNBC cells and reduced tumor growth compared with non-targeted formulations; exact numerical tumor-growth estimate NR
(Li et al., 2020)	Cellular uptake, viability and chemo-photothermal response	Combined NIR irradiation and magnetic targeting enhanced DOX delivery and reduced viability of MDR/MCF-7 cells relative to non-activated treatment; numerical effect estimate not extractable from the published report
(Tran et al., 2022)	Mitochondrial delivery and cytotoxicity	The formulation inhibited HeLa and SH-SY5Y viability while showing lower toxicity toward HEK293 cells; empty carrier showed an IC_50_ > 100 μg/mL in non-malignant cells
(Luo et al., 2019)	BBB transport, glioblastoma-cell uptake and cytotoxicity	Transferrin functionalization increased BBB transport and uptake by U87 glioblastoma cells relative to non-targeted pSiNPs; exact numerical cytotoxicity difference NR
(Zeng et al., 2019)	Cell viability, tumor growth, stemness and hypoxia markers	TMZ/PSi + PTT + HBO produced the greatest tumor-growth inhibition and reduced SOX2, Nestin and hypoxia-associated markers compared with single-component treatments; exact numerical percentage NR
(Xie et al., 2018)	Cell viability, cell-cycle distribution and tumor growth	TMZ/PSi + HBO produced an 84.2% tumor-inhibition rate, decreased hypoxia-induced C6 viability and promoted G2/M cell-cycle arrest

BBB: blood–brain barrier; CPT: camptothecin; DOX: doxorubicin; HBO: hyperbaric oxygen; HER2: human epidermal growth factor receptor 2; IC_50_: half-maximal inhibitory concentration; MDR: multidrug resistance; MRP1: multidrug resistance-associated protein 1; MTX: methotrexate; NIR: near-infrared; NR: indicates that the exact value was not reported in an extractable numerical form in the primary article; it does not indicate that an available numerical value was omitted during review extraction; PSi: porous silicon; pSiNPs: porous silicon nanoparticles; PTT: photothermal therapy; SOX2: SRY-box, transcription factor 2; TMZ: temozolomide; TNBC: triple-negative breast cancer.

### 3.3 Effects of drug-loaded pSiNPs in in vitro cancer models

All included studies reported at least one quantitative or comparative *in vitro* outcomes relevant to antitumor activity, drug delivery, or therapeutic selectivity. Drug-loaded pSiNPs generally demonstrated greater cytotoxicity or antiproliferative activity than empty particles and, in several studies, greater activity than the corresponding free therapeutic agent (Zhang et al., 2022; Correia et al., 2015; Abdelmoneem et al., 2025; Shahbazi et al., 2015; Konoplyannikov et al., 2021; Shahbazi et al., 2014; Shin et al., 2024; Wang et al., 2015; Luo et al., 2023; Landgraf et al., 2020; Tieu et al., 2021; Xia et al., 2018; Oaxaca Camacho et al., 2025; Zhang et al., 2017; Liu et al., 2017; Xue et al., 2022; Malhotra et al., 2025; Li et al., 2020; Tran et al., 2022; Luo et al., 2019; Zeng et al., 2019; Xie et al., 2018). These effects were particularly evident when pSiNPs were functionalized with targeting ligands or combined with stimulus-responsive mechanisms.

Comparator groups were classified hierarchically as untreated/vehicle control, free drug, empty pSiNPs, non-targeted drug-loaded pSiNPs, and targeted or multifunctional drug-loaded pSiNPs. This framework was used to distinguish the intrinsic effect of the porous-silicon carrier from the effect of drug loading and the incremental contribution of active targeting or other surface modifications (Zhang et al., 2022; Correia et al., 2015; Abdelmoneem et al., 2025; Shahbazi et al., 2015; Konoplyannikov et al., 2021; Shahbazi et al., 2014; Shin et al., 2024; Wang et al., 2015; Luo et al., 2023; Landgraf et al., 2020; Tieu et al., 2021; Xia et al., 2018; Oaxaca Camacho et al., 2025; Zhang et al., 2017; Liu et al., 2017; Xue et al., 2022; Malhotra et al., 2025; Li et al., 2020; Tran et al., 2022; Luo et al., 2019; Zeng et al., 2019; Xie et al., 2018).

For interpretation of formulation-performance relationships, particle size, polydispersity index, zeta potential, porosity, drug-loading capacity, and encapsulation efficiency were considered alongside cellular uptake, cytotoxicity, and *in vivo* tumor outcomes. Because these formulation variables were strongly confounded by payload, coating, targeting ligand, dose, cancer model, and assay conditions, the evidence did not support a valid cross-study correlation analysis. Accordingly, associations were interpreted only within directly comparable formulations from the same study (Zhang et al., 2022; Correia et al., 2015; Abdelmoneem et al., 2025; Shahbazi et al., 2015; Konoplyannikov et al., 2021; Shahbazi et al., 2014; Shin et al., 2024; Wang et al., 2015; Luo et al., 2023; Landgraf et al., 2020; Tieu et al., 2021; Xia et al., 2018; Oaxaca Camacho et al., 2025; Zhang et al., 2017; Liu et al., 2017; Xue et al., 2022; Malhotra et al., 2025; Li et al., 2020; Tran et al., 2022; Luo et al., 2019; Zeng et al., 2019; Xie et al., 2018).

Camptothecin-loaded pSiNPs with peptide-mediated targeting showed enhanced cytotoxicity in prostate cancer cells grown in both two-dimensional culture and three-dimensional spheroids (Zhang et al., 2022; Landgraf et al., 2020). Similarly, antibody-functionalized or receptor-responsive formulations carrying sorafenib, methotrexate, or doxorubicin improved cellular association and reduced cancer-cell viability relative to non-targeted formulations or free-drug controls (Correia et al., 2015; Shahbazi et al., 2015; Shahbazi et al., 2014; Wang et al., 2015; Oaxaca Camacho et al., 2025; Zhang et al., 2017). Targeted doxorubicin-loaded pSiNPs also showed selective toxicity in HER2-positive and triple-negative breast cancer cells (Oaxaca Camacho et al., 2025; Malhotra et al., 2025).

Several studies addressed multidrug resistance. Co-delivery systems combining doxorubicin with siRNA, photothermal dyes, iron oxide/gold nanocomponents, or quercetin-based surface modification enhanced intracellular drug delivery and reduced viability in resistant breast cancer or glioblastoma models (Tieu et al., 2021; Xia et al., 2018; Liu et al., 2017; Li et al., 2020; Tran et al., 2022). Improved therapeutic activity was associated with increased cellular uptake, endosomal escape, nuclear drug accumulation, controlled release under acidic conditions, or near-infrared irradiation.

Drug–nucleic acid co-delivery systems were retained only when they included an anticancer drug. In these formulations, the combination of temozolomide or doxorubicin with antisense oligonucleotides or siRNA enhanced the antiproliferative effect relative to single-component treatment strategies (Luo et al., 2023; Tieu et al., 2021). Studies evaluating nucleic acid therapy as the sole therapeutic intervention were excluded from this review.

### 3.4 Effects in in vivo cancer models

Eight studies evaluated drug-loaded pSiNPs in animal tumor models, including glioblastoma, breast cancer bone metastasis, Lewis lung carcinoma, and other xenograft or syngeneic tumor models (Konoplyannikov et al., 2021; Shin et al., 2024; Luo et al., 2023; Landgraf et al., 2020; Xue et al., 2022; Malhotra et al., 2025; Zeng et al., 2019; Xie et al., 2018). Within these individual experiments, pSiNP formulations were associated with lower tumor burden, reduced tumor growth or metastatic involvement, and, where assessed, longer survival than study-specific comparators. These findings provide preliminary *in vivo* evidence of antitumor activity but should not be interpreted as confirmation of therapeutic superiority.

Temozolomide-loaded pSiNPs were among the most extensively evaluated formulations in glioblastoma. Surface-modified formulations improved temozolomide delivery and therapeutic efficacy in intracranial glioblastoma models, resulting in lower tumor burden and longer survival than free temozolomide (Shin et al., 2024; Luo et al., 2023; Zeng et al., 2019; Xie et al., 2018). Combination approaches incorporating hyperbaric oxygen or mild photothermal therapy further enhanced antitumor effects in C6 glioma-bearing mice and were associated with modulation of stemness- and hypoxia-related markers (Zeng et al., 2019; Xie et al., 2018).

Camptothecin-loaded targeted pSiNPs reduced primary tumor growth and metastatic burden in a humanized breast cancer bone-metastasis model (Landgraf et al., 2020). Salinomycin-loaded mesoporous silicon nanoparticles also suppressed Lewis lung carcinoma growth following intraperitoneal administration (Konoplyannikov et al., 2021). In addition, targeted drug-loaded formulations showed antitumor activity in breast cancer models, supporting the contribution of surface targeting and stimulus-responsive release to *in vivo* therapeutic performance (Xue et al., 2022; Malhotra et al., 2025).

Collectively, these findings support the preclinical potential of drug-loaded pSiNP platforms to enhance drug delivery and antitumor activity under specific experimental conditions; although they do not establish clinical effectiveness.

### 3.5 Safety, biodistribution, and drug-delivery performance

Empty pSiNPs were generally reported to have low cytotoxicity at the tested concentrations in the *in vitro* systems evaluated (Zhang et al., 2022; Correia et al., 2015; Abdelmoneem et al., 2025; Shahbazi et al., 2015; Konoplyannikov et al., 2021; Shahbazi et al., 2014; Shin et al., 2024; Wang et al., 2015; Luo et al., 2023; Landgraf et al., 2020; Tieu et al., 2021; Xia et al., 2018; Oaxaca Camacho et al., 2025; Zhang et al., 2017; Liu et al., 2017; Xue et al., 2022; Malhotra et al., 2025; Li et al., 2020; Tran et al., 2022; Luo et al., 2019; Zeng et al., 2019; Xie et al., 2018). Where evaluated in animal studies, drug-loaded pSiNP formulations reported stable body weight, limited short-term systemic toxicity, or no overt histopathological abnormalities in the organs examined (Konoplyannikov et al., 2021; Shin et al., 2024; Landgraf et al., 2020; Xue et al., 2022; Malhotra et al., 2025; Zeng et al., 2019; Xie et al., 2018). Consequently, the available evidence is insufficient to establish systemic toxicity, chronic toxicity, long-term biocompatibility, or delayed effects of pSiNP degradation.

Targeting and surface-functionalization strategies generally enhanced cell-specific association, tumor accumulation, blood–brain barrier penetration, or intracellular drug delivery. Transferrin-functionalized pSiNPs improved doxorubicin delivery across an *in vitro* blood–brain barrier model and increased cytotoxicity toward glioblastoma cells (Luo et al., 2019). Other formulations improved tumor-cell uptake through antibody-, aptamer-, peptide-, nanobody-, or receptor-mediated interactions (Zhang et al., 2022; Shahbazi et al., 2015; Tieu et al., 2021; Oaxaca Camacho et al., 2025; Malhotra et al., 2025). Stimulus-responsive systems enabled pH-, redox-, or near-infrared-triggered drug release, with the aim of reducing premature release and increasing intracellular drug availability (Shahbazi et al., 2014; Xia et al., 2018; Xue et al., 2022; Li et al., 2020; Tran et al., 2022).

### 3.6 Methodological quality and risk of bias

The *in vitro* reliability appraisal indicated that most studies provided physicochemical characterization of the pSiNP formulations, included at least one comparator, and used established assays for cell viability, proliferation, cytotoxicity, or uptake. However, several reports did not clearly describe cell-line authentication, *mycoplasma* testing, allocation of experimental wells, blinding of outcome assessment, or the distinction between biological and technical replicates. Therefore, most *in vitro* studies were classified as reliable with restrictions (Table 4). These categories describe reliability and reporting adequacy and should not be interpreted as risk-of-bias ratings.

**TABLE 4:** *In vitro* methodological reliability appraisal using the adapted ToxRTool criteria.

Ref.	Group I: Test substance identification (0–3)	Group II: Test system characterization (0–3)	Group III: Study design description (0–4)	Group IV: Study results documentation (0–4)	Group V: Plausibility of study design and data (0–4)	Total (0–18)
(Zhang et al., 2022)	3	3	3	3	3	15
(Correia et al., 2015)	3	2	3	3	2	13
(Abdelmoneem et al., 2025)	3	3	3	3	3	15
(Shahbazi et al., 2015)	3	2	3	3	2	13
(Konoplyannikov et al., 2021)	3	2	3	2	2	12
(Shahbazi et al., 2014)	3	2	3	2	2	12
(Shin et al., 2024)	3	3	3	3	3	15
(Wang et al., 2015)	3	2	3	3	2	13
(Luo et al., 2023)	3	3	3	3	3	15
(Landgraf et al., 2020)	3	3	3	3	3	15
(Tieu et al., 2021)	3	2	3	3	3	14
(Xia et al., 2018)	3	2	3	2	2	12
(Oaxaca Camacho et al., 2025)	3	2	3	2	2	12
(Zhang et al., 2017)	3	2	3	3	2	13
(Liu et al., 2017)	3	2	3	3	2	13
(Xue et al., 2022)	3	3	3	3	3	15
(Malhotra et al., 2025)	3	3	3	3	3	15
(Li et al., 2020)	3	2	3	3	2	13
(Tran et al., 2022)	3	2	3	2	2	12
(Luo et al., 2019)	3	3	3	3	3	15
(Zeng et al., 2019)	3	2	3	3	2	13
(Xie et al., 2018)	3	2	3	2	2	12

Scoring: reliable without restrictions, 15–18 points; reliable with restrictions, 11–14 points; not reliable, ≤10 points. The adapted tool assessed reporting of test-substance identification, test-system characterization, study design, results documentation, and plausibility of the design and data.

For *in vivo* studies, risk of bias was assessed separately using SYRCLE. The principal concern was incomplete reporting of safeguards against bias. Selective reporting was judged low risk in all eight *in vivo* studies (8/8, 100%), and incomplete outcome data were generally judged low risk (7/8, 87.5%). Random sequence generation was rated low risk in four studies (4/8, 50.0%) and unclear in the remaining four. Baseline group similarity, allocation concealment, random housing, blinding of caregivers/investigators, random outcome assessment, blinding of outcome assessors, and other potential sources of bias were predominantly rated unclear because the relevant procedures were insufficiently reported (Figure 2). An unclear judgment indicates insufficient information and does not demonstrate that the safeguard was absent; nevertheless, it limits confidence in the estimated treatment effects.

**FIGURE 2:**
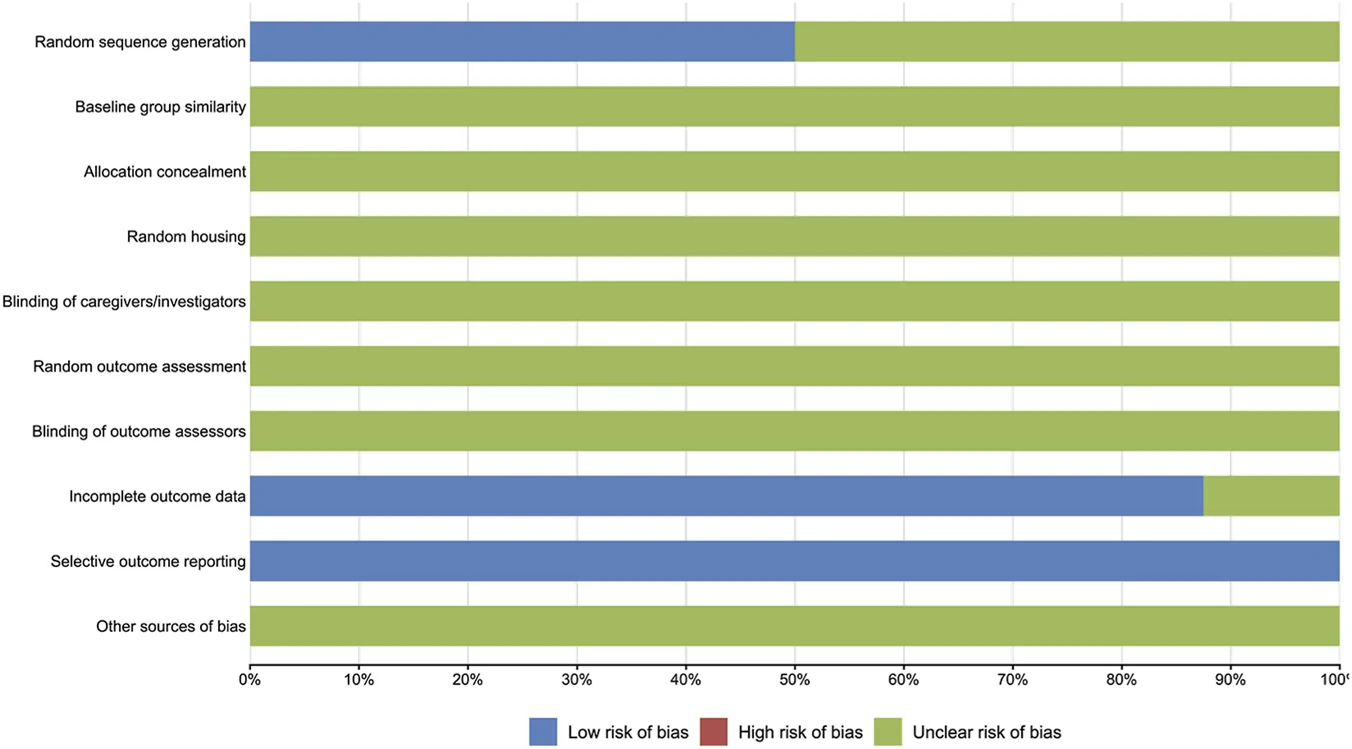
The risk of bias assessment of included studies based on SYRCLE risk of bias tool (Konoplyannikov et al., 2021; Shin et al., 2024; Luo et al., 2023; Landgraf et al., 2020; Xue et al., 2022; Malhotra et al., 2025; Zeng et al., 2019; Xie et al., 2018).

## 4 Discussion

### 4.1 Summary of key findings and interpretation

This systematic review synthesized 22 preclinical studies evaluating drug-loaded pSiNP platforms for cancer treatment. The available evidence consistently showed that pSiNPs can function as versatile reservoirs for conventional anticancer drugs and can be engineered to improve cellular uptake, tumor selectivity, intracellular trafficking, and stimulus-responsive release (Zhang et al., 2022; Correia et al., 2015; Abdelmoneem et al., 2025; Shahbazi et al., 2015; Konoplyannikov et al., 2021; Shahbazi et al., 2014; Shin et al., 2024; Wang et al., 2015; Luo et al., 2023; Landgraf et al., 2020; Tieu et al., 2021; Xia et al., 2018; Oaxaca Camacho et al., 2025; Zhang et al., 2017; Liu et al., 2017; Xue et al., 2022; Malhotra et al., 2025; Li et al., 2020; Tran et al., 2022; Luo et al., 2019; Zeng et al., 2019; Xie et al., 2018). In most studies, drug-loaded formulations produced greater cytotoxicity or antiproliferative activity than empty nanoparticles and, in several cases, outperformed the corresponding free drug or non-targeted formulation (Zhang et al., 2022; Correia et al., 2015; Abdelmoneem et al., 2025; Shahbazi et al., 2015; Shahbazi et al., 2014; Shin et al., 2024; Wang et al., 2015; Luo et al., 2023; Landgraf et al., 2020; Tieu et al., 2021; Xia et al., 2018; Oaxaca Camacho et al., 2025; Zhang et al., 2017; Liu et al., 2017; Xue et al., 2022; Malhotra et al., 2025; Li et al., 2020; Tran et al., 2022; Zeng et al., 2019). Eight studies extended their evaluation to animal models and generally reported inhibition of tumor growth, reduced metastatic burden, improved brain delivery, or prolonged survival (Konoplyannikov et al., 2021; Shin et al., 2024; Luo et al., 2023; Landgraf et al., 2020; Xue et al., 2022; Malhotra et al., 2025; Zeng et al., 2019; Xie et al., 2018).

Nevertheless, these findings should be interpreted as evidence of preclinical potential rather than proof of therapeutic superiority. The included studies differed substantially in nanoparticle composition, particle size, pore characteristics, surface chemistry, drug-loading method, release mechanism, administered dose, cancer model, exposure time, comparator, and outcome measurement (Zhang et al., 2022; Correia et al., 2015; Abdelmoneem et al., 2025; Shahbazi et al., 2015; Konoplyannikov et al., 2021; Shahbazi et al., 2014; Shin et al., 2024; Wang et al., 2015; Luo et al., 2023; Landgraf et al., 2020; Tieu et al., 2021; Xia et al., 2018; Oaxaca Camacho et al., 2025; Zhang et al., 2017; Liu et al., 2017; Xue et al., 2022; Malhotra et al., 2025; Li et al., 2020; Tran et al., 2022; Luo et al., 2019; Zeng et al., 2019; Xie et al., 2018). This methodological and biological heterogeneity prevented direct quantitative comparison and did not support a meaningful meta-analysis. Accordingly, the review provides a structured qualitative synthesis of the direction and consistency of the reported effects.

### 4.2 Therapeutic performance in in vitro cancer models

*In vitro* data indicate that encapsulation or loading of anticancer medications in pSiNPs may improve the apparent preclinical efficacy of these agents by enhancing their interaction with the cancer cells and changing their release profile. Camptothecin-loaded platforms increased the cytotoxicity in prostate and breast cancer models, including 2D cultures and 3D spheroids (Zhang et al., 2022; Landgraf et al., 2020). Dual-drug systems loaded with sorafenib, and methotrexate supported sustained release and combination therapy, while sorafenib-laden formulations reduced breast cancer-cell proliferation (Correia et al., 2015; Shahbazi et al., 2015; Wang et al., 2015). Likewise, methotrexate-loaded pSiNPs with zwitterionic or receptor-responsive surface alterations showed better cellular trafficking and therapeutic efficacy (Shahbazi et al., 2014; Zhang et al., 2017).

Doxorubicin was the most extensively investigated payload. Its frequent use may be explained by its broad anticancer activity, intrinsic fluorescence, and suitability for monitoring intracellular distribution. Doxorubicin-loaded pSiNPs showed enhanced uptake and cytotoxicity in osteosarcoma, HER2-positive breast cancer, multidrug-resistant breast cancer, triple-negative breast cancer, and glioblastoma models (Abdelmoneem et al., 2025; Tieu et al., 2021; Oaxaca Camacho et al., 2025; Liu et al., 2017; Xue et al., 2022; Malhotra et al., 2025; Li et al., 2020; Zeng et al., 2019). However, the magnitude of benefit varied according to the surface modification, cellular target, and release mechanism. This indicates that the presence of pSiNPs alone may not be sufficient to improve efficacy; the biological performance of the final platform depends on the interaction between the porous carrier, therapeutic cargo, surface coating, and target-cell phenotype.

Most studies compared drug-loaded pSiNPs with empty particles, free drug, or non-targeted nanoparticles. These comparators are important because they help distinguish the intrinsic cytotoxicity of the carrier from the activity of the loaded drug and the added value of targeting. Nevertheless, comparator selection was not standardized. Some studies lacked direct comparisons with clinically relevant formulations or equivalent drug concentrations, and differences in exposure time and dose normalization complicated comparisons across platforms (Zhang et al., 2022; Correia et al., 2015; Abdelmoneem et al., 2025; Shahbazi et al., 2015; Konoplyannikov et al., 2021; Shahbazi et al., 2014; Shin et al., 2024; Wang et al., 2015; Luo et al., 2023; Landgraf et al., 2020; Tieu et al., 2021; Xia et al., 2018; Oaxaca Camacho et al., 2025; Zhang et al., 2017; Liu et al., 2017; Xue et al., 2022; Malhotra et al., 2025; Li et al., 2020; Tran et al., 2022; Luo et al., 2019; Zeng et al., 2019; Xie et al., 2018). Future *in vitro* studies should therefore report drug-equivalent concentrations and include empty particles, free drug, non-targeted drug-loaded particles, and targeted drug-loaded particles as separate experimental groups.

### 4.3 Contribution of surface functionalization and active targeting

Surface engineering was a defining feature of the included pSiNP platforms. The strategies included cRGDfK peptides, alendronate, CD326-and HER2-directed antibodies, nanobodies, aptamers, transferrin, quercetin derivatives, polyethylene glycol, polyHPAm, and zwitterionic polymer coatings (Zhang et al., 2022; Abdelmoneem et al., 2025; Shahbazi et al., 2015; Shahbazi et al., 2014; Tieu et al., 2021; Oaxaca Camacho et al., 2025; Liu et al., 2017; Malhotra et al., 2025; Luo et al., 2019). These modifications were aimed to improve colloidal stability, lower non-specific interactions, recognize tumor-associated receptors, promote internalization, facilitate endosomal escape, or help target the nanoparticles in certain tissues.

The results imply that active targeting can enhance cell-specific interaction and intracellular medication delivery. Interactions of CD326-mediated pSiNPs with receptor-bearing cancer cells and the HER2-targeted particles demonstrated selective activity in HER2 + breast cancer models (Shahbazi et al., 2015; Oaxaca Camacho et al., 2025). Aptamer functionalized pSiNPs enhanced the delivery to triple negative breast cancer cells (Malhotra et al., 2025), and alendronate modified particles were intended to boost the affinity of doxorubicin-loaded nanoparticles with bone and osteosarcoma associated targets (Abdelmoneem et al., 2025). Transferrin-functionalized pSiNPs also increased doxorubicin distribution across an *in vitro* blood-brain barrier model as well as boosted uptake by glioma cells (Luo et al., 2019).

These data indicate active targeting as a viable technique, but expression of receptors in experimental cell lines is usually better regulated than in heterogeneous human cancers. Target expression may change between patients, between primary and metastatic lesions, and during the course of treatment. Furthermore, higher cellular uptake *in vitro* may not necessarily translate to enhanced tumor accumulation *in vivo*, where protein adsorption, immunological clearance, vascular permeability and nanoparticle distribution can all impact targeted efficacy. Therefore, more research should explore target expression in varied tumor models and show that the targeting is efficient in biologically complicated *in vivo* conditions.

### 4.4 Stimulus-responsive release and combination therapy

Drug-loading capacity, encapsulation efficiency, and release behavior were evaluated as formulation-specific determinants of therapeutic performance. Release data were interpreted together with the experimental medium, pH, stimulus, and sampling duration because cumulative release percentages obtained under different conditions are not directly interchangeable. Where loading or encapsulation values were not reported, this was retained as a reporting gap rather than estimated from other formulation data (Zhang et al., 2022; Correia et al., 2015; Abdelmoneem et al., 2025; Shahbazi et al., 2015; Konoplyannikov et al., 2021; Shahbazi et al., 2014; Shin et al., 2024; Wang et al., 2015; Luo et al., 2023; Landgraf et al., 2020; Tieu et al., 2021; Xia et al., 2018; Oaxaca Camacho et al., 2025; Zhang et al., 2017; Liu et al., 2017; Xue et al., 2022; Malhotra et al., 2025; Li et al., 2020; Tran et al., 2022; Luo et al., 2019; Zeng et al., 2019; Xie et al., 2018).

Several platforms were designed to release their payload in response to acidic conditions, redox changes, near-infrared irradiation, magnetic fields, or combinations of these stimuli (Xia et al., 2018; Xue et al., 2022; Li et al., 2020; Tran et al., 2022; Luo et al., 2019). Such systems aim to limit premature drug release during circulation and increase drug availability within the tumor microenvironment or intracellular compartments. Near-infrared-responsive platforms combined drug delivery with photothermal activity, while graphene oxide and iron oxide/gold-containing hybrid systems added optical or magnetic responsiveness to the porous silicon carrier (Xia et al., 2018; Li et al., 2020; Tran et al., 2022).

The reviewed studies generally reported stronger cytotoxic or antitumor effects when chemotherapy was combined with photothermal treatment than when either component was applied alone (Xia et al., 2018; Xue et al., 2022; Li et al., 2020; Tran et al., 2022; Zeng et al., 2019). Nevertheless, multifunctionality also increases formulation complexity. Each additional component may alter particle size, surface charge, colloidal stability, biodegradation, pharmacokinetics, and toxicity. It can also create challenges for reproducible manufacturing and regulatory characterization. Therefore, the benefit of multifunctional pSiNPs should be demonstrated against simpler formulations to determine whether the additional components provide a clinically meaningful advantage.

For synthesis, multifunctional systems were grouped according to their principal engineering strategy: drug-only pSiNPs, actively targeted systems, stimulus-responsive systems, chemo-photothermal systems, magnetic or hybrid systems, and drug–nucleic-acid co-delivery systems. Incremental benefit was attributed to multifunctionality only when a simpler, otherwise comparable formulation was included as a direct control; heterogeneous multifunctional platforms were not pooled as a single quantitative category (Luo et al., 2023; Tieu et al., 2021; Xia et al., 2018; Xue et al., 2022; Li et al., 2020; Tran et al., 2022; Zeng et al., 2019; Xie et al., 2018).

Drug–nucleic acid co-delivery represented another combination strategy. Doxorubicin–siRNA and temozolomide–antisense oligonucleotide systems were retained because they included a conventional anticancer drug in addition to the nucleic acid cargo (Luo et al., 2023; Tieu et al., 2021). These formulations showed that pSiNPs can accommodate agents with different physicochemical properties and support simultaneous chemotherapy and gene modulation. However, studies evaluating siRNA or anti-miR therapy as the sole intervention were excluded according to the eligibility criteria. The conclusions of this review should therefore be restricted to drug-loaded platforms and should not be generalized to pSiNP-based gene therapy without an anticancer drug.

### 4.5 Glioblastoma and the challenge of brain delivery

Glioblastoma was one of the most frequently investigated malignancies. This emphasis is relevant because inadequate penetration across the blood–brain barrier, diffuse tumor invasion, treatment resistance, hypoxia, and glioma stem-like cells substantially limit conventional therapy. Temozolomide-loaded pSiNPs improved drug stability, brain accumulation, or antitumor activity in glioblastoma models (Shin et al., 2024; Luo et al., 2023; Zeng et al., 2019; Xie et al., 2018). Transferrin-functionalized doxorubicin-loaded particles also showed improved transport across a blood–brain barrier model and enhanced uptake by glioblastoma cells (Luo et al., 2019).

Combination strategies involving temozolomide-loaded pSiNPs, hyperbaric oxygen, and mild photothermal treatment produced stronger antitumor effects in C6 glioma models and were associated with changes in hypoxia- and stemness-related markers (Zeng et al., 2019; Xie et al., 2018). These findings suggest that pSiNP platforms may support simultaneous intervention against drug delivery limitations and biological mechanisms of resistance. However, the C6 glioma model, subcutaneous tumors, and simplified blood–brain barrier systems do not reproduce all features of human glioblastoma. Validation in patient-derived glioma cells, organoids, immunocompetent models, and orthotopic tumors with infiltrative growth would strengthen the translational relevance of these findings.

### 4.6 Evidence from animal tumor models

Eight studies evaluated drug-loaded pSiNPs in animal models (Konoplyannikov et al., 2021; Shin et al., 2024; Luo et al., 2023; Landgraf et al., 2020; Xue et al., 2022; Malhotra et al., 2025; Zeng et al., 2019; Xie et al., 2018). Salinomycin-loaded mesoporous silicon nanoparticles suppressed Lewis lung carcinoma growth (Konoplyannikov et al., 2021), while targeted camptothecin-loaded particles reduced primary tumor growth and metastatic involvement in a humanized breast cancer bone-metastasis model (Landgraf et al., 2020). Doxorubicin-loaded, stimulus-responsive or aptamer-targeted formulations also demonstrated antitumor activity in cervical cancer and triple-negative breast cancer xenografts (Xue et al., 2022; Malhotra et al., 2025). In glioma models, temozolomide-loaded pSiNPs reduced tumor burden or improved survival, particularly when combined with surface modification, antisense oligonucleotides, hyperbaric oxygen, or photothermal treatment (Shin et al., 2024; Luo et al., 2023; Zeng et al., 2019; Xie et al., 2018).

Although the direction of these findings was generally favorable, the animal evidence remains limited. Most experiments involved relatively small groups, short observation periods, and a single tumor model, and several used immunodeficient animals. In addition, the predominance of unclear SYRCLE judgments for randomization, allocation concealment, housing, and blinding reduces confidence in the magnitude of the reported effects. The animal findings should therefore be considered preliminary rather than confirmatory. Replication in adequately powered, independently conducted, complementary animal models is required before clinical translation can be considered.

### 4.7 Safety, biocompatibility, and biodistribution

Empty pSiNPs generally showed low cytotoxicity at the concentrations evaluated in the included *in vitro* studies (Zhang et al., 2022; Correia et al., 2015; Abdelmoneem et al., 2025; Shahbazi et al., 2015; Konoplyannikov et al., 2021; Shahbazi et al., 2014; Shin et al., 2024; Wang et al., 2015; Luo et al., 2023; Landgraf et al., 2020; Tieu et al., 2021; Xia et al., 2018; Oaxaca Camacho et al., 2025; Zhang et al., 2017; Liu et al., 2017; Xue et al., 2022; Malhotra et al., 2025; Li et al., 2020; Tran et al., 2022; Luo et al., 2019; Zeng et al., 2019; Xie et al., 2018). Animal studies frequently reported stable body weight, limited short-term systemic toxicity, or no evident histopathological alterations in the organs examined (Konoplyannikov et al., 2021; Shin et al., 2024; Landgraf et al., 2020; Xue et al., 2022; Malhotra et al., 2025; Zeng et al., 2019; Xie et al., 2018). These observations are encouraging but constitute only preliminary tolerability signals, not evidence of established systemic safety or long-term biocompatibility.

Only eight included studies contained animal experiments, often with small groups and short follow-up. Most emphasized short-term tolerability and did not systematically assess hematological toxicity, hepatic and renal function, immune activation, inflammatory responses, reproductive toxicity, chronic tissue accumulation, or delayed effects of nanoparticle degradation. Biodistribution analyses were inconsistent, and relatively few studies quantified nanoparticle or drug concentrations across tumors and major organs over time (Shin et al., 2024; Luo et al., 2023; Landgraf et al., 2020; Malhotra et al., 2025). Therefore, conclusions regarding systemic toxicity, chronic toxicity, long-term biocompatibility, and delayed degradation-related effects remain speculative. The absence of overt toxicity during a short experiment must not be interpreted as evidence of long-term safety.

Safety assessment should also consider the complete formulation rather than porous silicon alone. Polymer coatings, targeting ligands, graphene oxide, photothermal agents, iron oxide, gold components, and residual manufacturing reagents may modify the biological response (Tieu et al., 2021; Xia et al., 2018; Xue et al., 2022; Li et al., 2020; Tran et al., 2022). Future studies should characterize the degradation and clearance of each component, establish maximum tolerated doses, and compare systemic toxicity with that of the corresponding free drug.

### 4.8 Methodological quality and certainty of the evidence

The adapted ToxRTool appraisal indicated that most *in vitro* studies were reliable with restrictions. This appraisal addresses methodological reliability and reporting adequacy, not risk of bias. Strengths included physicochemical characterization, relevant comparators, drug-release experiments, and established assays for viability, uptake, proliferation, or cytotoxicity. Frequent limitations were insufficient reporting of cell-line authentication, *mycoplasma* testing, allocation of experimental wells, biological versus technical replicates, and blinding of outcome assessment. Because the adaptation of ToxRTool for nanomedicine efficacy experiments has not been independently validated, these classifications should be interpreted descriptively.

The SYRCLE assessment showed that incomplete reporting was also a major limitation of the animal evidence. Random sequence generation was clearly reported in only some studies, while allocation concealment, random housing, caregiver blinding, random outcome assessment, and assessor blinding were generally unclear (Konoplyannikov et al., 2021; Shin et al., 2024; Luo et al., 2023; Landgraf et al., 2020; Xue et al., 2022; Malhotra et al., 2025; Zeng et al., 2019; Xie et al., 2018). Incomplete reporting does not demonstrate that these procedures were omitted, but it prevents a confident judgment of low risk of bias. Because tumor measurement and histological interpretation can be influenced by knowledge of group allocation, blinded assessment is particularly important.

The predominance of unclear judgments reduces confidence in the magnitude of the reported therapeutic effects. Future animal studies should follow established reporting guidance, prospectively define primary outcomes, justify sample sizes, describe randomization procedures, document exclusions and attrition, and report all prespecified outcomes. These measures would improve reproducibility and permit a more reliable estimation of therapeutic efficacy.

### 4.9 Strengths and limitations

A strength of this review was the use of tight qualifying criteria that particularly targeted drug-loaded pSiNPs tested for cancer treatment with quantified *in vitro* or *in vivo* evidence. This technique excludes imaging-only platforms, characterization or release only research, mesoporous silica systems, conference abstracts and nucleic acid therapy without an anticancer agent. Therefore, the synthesis is directly related to the therapeutic performance of porous silicon platforms loaded with drugs.

The review separately appraised *in vitro* reliability with adapted ToxRTool criteria and animal risk of bias with SYRCLE. This distinction was necessary because experimental cell studies and animal studies are affected by different sources of bias. Both assessments depended on information reported in the primary articles; procedures that were performed but not reported could not be credited, and unclear SYRCLE judgments should therefore be interpreted as uncertainty rather than evidence that a safeguard was absent.

Several limitations should be acknowledged. First, substantial heterogeneity in cancer models, pSiNP formulations, therapeutic payloads, doses, exposure periods, comparators, and outcome measures precluded meta-analysis. Second, most evidence was derived from established cancer-cell lines, and only a minority of studies used three-dimensional cultures, blood–brain barrier models, patient-relevant systems, or orthotopic tumors. Third, publication bias and selective outcome reporting could not be formally evaluated. Finally, no clinical studies were identified, and the conclusions are therefore restricted to preclinical evidence.

A contextual distinction from mesoporous silica nanoparticles (MSNs) is also important. Both porous silicon and mesoporous silica platforms offer high internal surface area, tunable porosity, surface functionalization, and controlled-release opportunities, but they differ in framework chemistry, degradation behavior, and translational considerations. MSNs were not included in the systematic dataset because the eligibility criteria required porous elemental silicon as the drug-carrying phase; comparisons with the MSN literature are therefore contextual rather than part of the evidence synthesis (Yan et al., 2024).

### 4.10 Implications for future research and clinical translation

Future studies should address repeatability and clinically relevant comparisons. Report physicochemical properties such particle size and distribution, shape, porosity, surface area, surface charge, drug-loading capacity, encapsulation efficiency, release kinetics, colloidal stability, degradation and batch-to-batch variability. Drug dosages should be represented in terms of the active drug, and treatment comparisons should be made on the basis of equivalent levels of the active drug.

Experimental efficacy should be verified in multicellular spheroids, organoids, patient-derived cells, orthotopic or immunocompetent animal models. Studies should include comparisons of drug-loaded pSiNPs, free drug, empty pSiNPs, non-targeted drug-loaded pSiNPs and existing standard-of-care therapies. Targeted systems should experimentally demonstrate receptor expression and target dependence. For externally activated platforms, tissue penetration, duration, intensity and safety of near-infrared irradiation or magnetic stimulation should be described (Xia et al., 2018; Xue et al., 2022; Li et al., 2020; Tran et al., 2022; Luo et al., 2019).

Long-term pharmacokinetic, biodistribution, degradation and toxicological studies are also necessary. The decision to translate will depend not only on antitumor activity but also on scalable production, sterilization, storage stability, repeatability, and the capacity to describe complicated multifunctional formulations. Where possible, simplifying nanoparticle design could aid in manufacturing and regulatory evaluation, without necessarily losing therapeutic efficacy.

Advanced manufacturing approaches, including three-dimensional printing, may also provide future opportunities for integrating nanoparticle-based delivery into personalized dosage forms or spatially controlled therapeutic systems. At present, however, such approaches should be regarded as a translational research direction rather than evidence supporting the efficacy of pSiNPs in cancer, because direct pSiNP-oncology data using 3D-printed dosage forms were not identified in this review (Auel et al., 2025).

A Quality-by-Design (QbD) framework could further improve reproducibility and scale-up of pSiNP formulations. Future development should prospectively define the quality target product profile and identify critical material attributes and critical process parameters affecting particle size, pore architecture, surface charge, drug loading, encapsulation efficiency, release kinetics, colloidal stability, and batch-to-batch variability. Design-of-experiments and response-surface approaches may be particularly useful for establishing multidimensional design spaces instead of optimizing one formulation variable at a time (Camacho Vieira et al., 2024).

To summarize, drug-loaded pSiNPs provide a promising and versatile platform for targeted cancer therapy, regulated drug release, reversal of drug resistance and multimodal treatment. The evidence is most robust at the *in vitro* proof-of-concept level, although positive, albeit very limited, findings from animal studies confirm this (Zhang et al., 2022; Correia et al., 2015; Abdelmoneem et al., 2025; Shahbazi et al., 2015; Konoplyannikov et al., 2021; Shahbazi et al., 2014; Shin et al., 2024; Wang et al., 2015; Luo et al., 2023; Landgraf et al., 2020; Tieu et al., 2021; Xia et al., 2018; Oaxaca Camacho et al., 2025; Zhang et al., 2017; Liu et al., 2017; Xue et al., 2022; Malhotra et al., 2025; Li et al., 2020; Tran et al., 2022; Luo et al., 2019; Zeng et al., 2019; Xie et al., 2018). Rigorous and standardized preclinical investigations are needed to assess whether the stated benefits in drug delivery translate into reproducible therapeutic advantages and an acceptable safety profile for clinical development.

In summary, drug-loaded pSiNPs represent a promising platform for targeted cancer drug delivery, controlled release, reversal of drug resistance, and multimodal treatment. The evidence is strongest at the *in vitro* proof-of-concept level. The eight studies containing animal experiments provide preliminary supportive evidence, but they do not confirm therapeutic superiority or long-term safety (Correia et al., 2015; Abdelmoneem et al., 2025; Shahbazi et al., 2015; Konoplyannikov et al., 2021; Shahbazi et al., 2014; Shin et al., 2024; Wang et al., 2015; Luo et al., 2023; Landgraf et al., 2020; Tieu et al., 2021; Xia et al., 2018; Oaxaca Camacho et al., 2025; Zhang et al., 2017; Liu et al., 2017; Xue et al., 2022; Malhotra et al., 2025; Li et al., 2020; Tran et al., 2022; Luo et al., 2019; Zeng et al., 2019; Xie et al., 2018; Yan et al., 2024). Therefore, rigorous, standardized, and adequately powered preclinical studies are needed to determine whether the observed drug-delivery advantages translate into reproducible antitumor efficacy and an acceptable safety profile.

## 5 Conclusion

Drug-loaded porous silicon nanoparticles represent a promising preclinical platform for cancer drug delivery. Across the 22 included studies, the evidence was predominantly derived from *in vitro* models and suggested improvements in intracellular delivery, controlled release, cancer-cell selectivity, and antitumor activity relative to study-specific comparators (Zhang et al., 2022; Correia et al., 2015; Abdelmoneem et al., 2025; Shahbazi et al., 2015; Konoplyannikov et al., 2021; Shahbazi et al., 2014; Shin et al., 2024; Wang et al., 2015; Luo et al., 2023; Landgraf et al., 2020; Tieu et al., 2021; Xia et al., 2018; Oaxaca Camacho et al., 2025; Zhang et al., 2017; Liu et al., 2017; Xue et al., 2022; Malhotra et al., 2025; Li et al., 2020; Tran et al., 2022; Luo et al., 2019; Zeng et al., 2019; Xie et al., 2018). Only eight studies included animal experiments; these provided preliminary evidence of reduced tumor growth or metastatic burden and, in some models, longer survival (Konoplyannikov et al., 2021; Shin et al., 2024; Luo et al., 2023; Landgraf et al., 2020; Xue et al., 2022; Malhotra et al., 2025; Zeng et al., 2019; Xie et al., 2018). However, small animal cohorts, short follow-up, heterogeneous formulations and models, incomplete safety assessment, and predominantly unclear risk-of-bias judgments in several SYRCLE domains substantially limit confidence in the magnitude and durability of these effects. Current evidence therefore does not establish systemic safety, long-term biocompatibility, or clinical effectiveness. Future work should use standardized nanoparticle characterization, clinically relevant and adequately powered animal models, appropriate comparators, randomization and blinded outcome assessment, and comprehensive pharmacokinetic, biodistribution, degradation, and long-term toxicology analyses before clinical translation is considered.

However, the evidence remains exclusively preclinical and is limited by substantial methodological heterogeneity, small animal cohorts, short follow-up periods, incomplete safety assessments, and unclear risk of bias in several domains. Future studies should incorporate standardized nanoparticle characterization, clinically relevant tumor models, appropriate comparators, adequately powered experiments, randomization, blinded outcome assessment, and comprehensive pharmacokinetic, biodistribution, degradation, and toxicity analyses. Addressing these limitations will be essential to determine whether the promising preclinical performance of drug-loaded pSiNPs can be translated into safe, reproducible, and clinically meaningful cancer therapies.

## Data Availability

The original contributions presented in the study are included in the article/Supplementary Material, further inquiries can be directed to the corresponding authors.
