# Supplementary material for: Preclinical efficacy and safety of drug-loaded porous silicon nanoparticles for cancer treatment: a systematic review of in vitro and in vivo studies
Source: Front Pharmacol. 2026 Sep 17;17:1969890. doi: 10.3389/fphar.2026.1969890 (PMC13628494; doi:10.3389/fphar.2026.1969890)
Supplement: Supplementary file 1 [file Table1.docx]

Supplementary Material

# 1 Supplementary Figures and Tables

**Supplementary Table 1.** Complete electronic search strategies used to identify studies evaluating drug-loaded porous silicon nanoparticles for cancer treatment.

| **Database** | **Search strategy** |
| --- | --- |
| Medline | ("porous silicon" OR "nanoporous silicon" OR "porous silicon nanoparticle*"  OR "porous silicon nanocarrier*" OR pSiNP*) AND ("drug load*" OR "drug deliver*"  OR nanocarrier* OR "controlled release" OR "targeted deliver*" OR "stimuli responsive" OR "stimulus responsive" OR "therapeutic agent*" OR therapeutics) AND (cancer* OR neoplasm* OR tumor* OR tumour* OR carcinoma* OR malignan* OR oncolog*) |
| Embase | ('porous silicon':ti,ab,kw OR 'nanoporous silicon':ti,ab,kw OR 'porous silicon nanoparticle':ti,ab,kw OR 'porous silicon nanoparticles':ti,ab,kw OR 'porous silicon nanocarrier':ti,ab,kw OR 'porous silicon nanocarriers':ti,ab,kw OR psinp:ti,ab,kw OR psinps:ti,ab,kw) AND ('drug loading':ti,ab,kw OR 'drug loaded':ti,ab,kw OR 'drug delivery':ti,ab,kw OR nanocarrier*:ti,ab,kw OR 'controlled release':ti,ab,kw OR 'targeted delivery':ti,ab,kw OR 'stimuli responsive':ti,ab,kw OR 'stimulus responsive':ti,ab,kw OR 'therapeutic agent':ti,ab,kw OR 'therapeutic agents':ti,ab,kw OR therapeutics:ti,ab,kw)  AND ('neoplasm'/exp OR cancer*:ti,ab,kw OR neoplasm*:ti,ab,kw OR tumor*:ti,ab,kw OR tumour*:ti,ab,kw OR carcinoma*:ti,ab,kw OR malignan*:ti,ab,kw OR oncolog*:ti,ab,kw) |
| Scopus | TITLE-ABS-KEY("porous silicon" OR "nanoporous silicon" OR "porous silicon nanoparticle" OR "porous silicon nanoparticles" OR "pSi nanoparticle" OR "pSi nanoparticles" OR pSiNP OR pSiNPs) AND TITLE-ABS-KEY("drug load*" OR "drug-load*" OR "drug deliver*" OR nanocarrier* OR "controlled release" OR "targeted deliver*" OR "stimuli-responsive" OR "stimuli responsive" OR "stimulus-responsive" OR "stimulus responsive" OR "therapeutic agent*") AND TITLE-ABS-KEY(cancer* OR neoplasm* OR tumor* OR tumour* OR carcinoma* OR malignan* OR oncolog*) AND PUBYEAR < 2026 |
| Web of Science Core Collection | TS=("porous silicon" OR "nanoporous silicon" OR "porous silicon nanoparticle*" OR "pSi nanoparticle*" OR pSiNP*) AND TS=("drug load*" OR "drug-load*" OR "drug deliver*" OR nanocarrier* OR "controlled release" OR "targeted deliver*" OR "stimuli-responsive" OR "stimuli responsive" OR "stimulus-responsive" OR "stimulus responsive" OR "therapeutic agent*") AND TS=(cancer* OR neoplasm* OR tumor* OR tumour* OR carcinoma* OR malignan* OR oncolog*) AND PY=(1900-2025) |
| International Pharmaceutical Abstracts | (TI ("porous silicon" OR "nanoporous silicon" OR "porous silicon nanoparticle*" OR "porous silicon nanocarrier*" OR pSiNP*) OR AB ("porous silicon" OR "nanoporous silicon" OR "porous silicon nanoparticle*" OR "porous silicon nanocarrier*" OR pSiNP*)) AND (TI ("drug load*" OR "drug deliver*" OR "drug encapsulat*" OR nanocarrier* OR "controlled release" OR "targeted deliver*" OR "stimuli responsive" OR "stimulus responsive" OR "therapeutic agent*" OR "anticancer agent*" OR chemotherap*) OR AB ("drug load*" OR "drug deliver*" OR "drug encapsulat*" OR nanocarrier* OR "controlled release" OR "targeted deliver*" OR "stimuli responsive" OR "stimulus responsive" OR "therapeutic agent*" OR "anticancer agent*" OR chemotherap*)) AND (TI (cancer* OR neoplasm* OR tumor* OR tumour* OR carcinoma* OR malignan* OR oncolog*) OR AB (cancer* OR neoplasm* OR tumor* OR tumour* OR carcinoma* OR malignan* OR oncolog*)) |
| Applied Science and Technology Source Ultimate | (TI ("porous silicon" OR "nanoporous silicon" OR "porous silicon nanoparticle*" OR "porous silicon nanocarrier*" OR "porous silicon nanostructure*" OR pSiNP*) OR AB ("porous silicon" OR "nanoporous silicon" OR "porous silicon nanoparticle*" OR "porous silicon nanocarrier*" OR "porous silicon nanostructure*" OR pSiNP*)) AND ( TI ("drug load*" OR "drug deliver*" OR "drug encapsulat*" OR nanocarrier* OR "controlled release" OR "targeted deliver*" OR "stimuli responsive" OR "stimulus responsive" OR functionaliz* OR "surface modif*" OR "therapeutic agent*" OR "anticancer agent*") OR AB ("drug load*" OR "drug deliver*" OR "drug encapsulat*" OR nanocarrier* OR "controlled release" OR "targeted deliver*" OR "stimuli responsive" OR "stimulus responsive" OR functionaliz* OR "surface modif*" OR "therapeutic agent*" OR "anticancer agent*")) AND (TI (cancer* OR neoplasm* OR tumor* OR tumour* OR carcinoma* OR malignan* OR oncolog* OR antitumor* OR antitumour*) OR AB (cancer* OR neoplasm* OR tumor* OR tumour* OR carcinoma* OR malignan* OR oncolog* OR antitumor* OR antitumour*)) |
